# Onset timing of statin‐induced musculoskeletal adverse events and concomitant drug‐associated shift in onset timing of MAEs

**DOI:** 10.1002/prp2.439

**Published:** 2018-11-07

**Authors:** Hayato Akimoto, Akio Negishi, Shinji Oshima, Mitsuyoshi Okita, Sachihiko Numajiri, Naoko Inoue, Shigeru Ohshima, Daisuke Kobayashi

**Affiliations:** ^1^ Department of Analytical Pharmaceutics and Informatics Faculty of Pharmacy and Pharmaceutical Sciences Josai University Sakado Saitama Japan; ^2^ Josai University Pharmacy Sakado Saitama Japan

**Keywords:** concomitant drug, drug‐drug interaction, musculoskeletal adverse event, onset timing, rhabdomyolysis, statin

## Abstract

To evaluate the onset timing of musculoskeletal adverse events (MAEs) that develop during statin monotherapy and to determine whether concomitant drugs used concurrently with statin therapy shifts the onset timing of MAEs. Cases in which statins (atorvastatin, rosuvastatin, simvastatin, lovastatin, fluvastatin, pitavastatin, and pravastatin) were prescribed were extracted from the US Food and Drug Administration (FDA) Adverse Event Reporting System (FAERS) Data Files. The onset timing of MAEs during statin monotherapy was evaluated by determining the difference between statin start date and MAE onset date. The use of concomitant drugs with statin therapy was included in the analysis. Statins used in combination with concomitant drugs were compared with statin monotherapy to determine if the use of concomitant drugs shifted the onset timing of MAEs. The onset of MAEs was significantly faster with atorvastatin and rosuvastatin than with simvastatin. A difference in onset timing was not detected with other statins because the number of cases was too small for analysis. When evaluating concomitant drug use, the concomitant drugs that shifted the onset timing of MAEs could not be detected. Statins with strong low‐density lipoprotein cholesterol‐lowering effects (atorvastatin and rosuvastatin) contributed not only to a high risk of MAE onset, but also to a shorter time‐to‐onset. No concomitant drug significantly shifted the onset timing of MAEs when used concurrently with statins.

**Abbreviations**
FAERS: Food and Adverse Event Reporting System
FDA: Food and Drug Administration
HMG‐CoA: 3‐hydroxy 3‐methylglutaryl CoA
LDL: low‐density lipoprotein
MAEs: musculoskeletal adverse events

## 1 INTRODUCTION

Statins are 3‐hydroxy 3‐methylglutaryl CoA (HMG‐CoA) reductase inhibitors and low‐density lipoprotein (LDL) cholesterol‐lowering agents. They are well‐tolerated and are known to lower the risk of atherosclerotic cardiovascular disease (ASCVD).^1^, ^2^ However, if musculoskeletal adverse events (MAEs) such as myalgia, myopathy, and rhabdomyolysis develop during the statin use period, these cholesterol‐lowering treatments may need to be temporarily or permanently discontinued.^2^ There are a few reports detailing the onset timing of drug‐induced adverse events.^3^, ^4^ In 2004, Chang et al reported the onset timing of statin‐induced rhabdomyolysis.^5^ However, the difference in onset timing of rhabdomyolysis between each statin was not detected owing to few number of cases.

It is difficult to detect drug‐drug interactions (DDIs) that may cause severe adverse events at the stage of drug approval examination.^6^ DDIs are usually discovered during nonmarketing surveillance.^7^, ^8^ It is already known that the concomitant use of statins and specific nonstatin drugs increases the risk of rhabdomyolysis. For example, the concomitant use of statins with fibrates^9^, ^10^, ^11^ or cytochrome P450 (CYP) inhibitors, such as clarithromycin (CYP3A4 inhibitor), cyclosporine (CYP3A4 inhibitor), and clopidogrel (CYP2C8 inhibitor), increases the risk of rhabdomyolysis.^12^, ^13^, ^14^ It has been reported that the increased risk of statin‐induced rhabdomyolysis may be due to the pharmacokinetic changes caused by concomitant drugs.^15^ If DDIs cause changes in the time‐course of blood concentration of statins, it not only changes the onset risk, but may also affect the onset timing. There are limited studies evaluating the risk of concomitant drugs on the onset timing of statin adverse events.

To reduce and prevent the risk of adverse events in a clinical setting, it is important to acquire information on both the risk and onset timing of drug‐induced adverse events. We have already evaluated the onset timing of adverse events as well as the risk of these events.^3^, ^16^ Although many drugs have the potential to cause the same adverse event, especially those within the same medication class, the onset timing of these events for individual drugs may differ; thus, it is important to evaluate the onset timing of side effects associated with each drug.

The Food and Drug Administration (FDA) Adverse Event Reporting System (FAERS) is often used to detect DDIs. Risk evaluation using disproportionality is performed to determine these DDIs.^17^, ^18^ Although the incidence of statin‐induced MAEs differs in literature, statins are well‐tolerated and rarely cause MAEs. Therefore, the incidence of statin‐induced MAEs is very low,^11^, ^19^, ^20^ and it is difficult to evaluate the onset timing of statin‐induced MAEs through clinical trials as these adverse events may occur within 12 months of starting statin therapy or after many years.^10^ Since clinical trials are performed only for a specific period, nonmarketing surveillance and reporting of adverse events through FAERS is helpful to determine the onset timing of MAEs. The FAERS is a large‐scale database that accumulates reported cases of adverse events; thus, it is suitable for analysing MAEs that develop at a low frequency during statin use.

Therefore, this study was aimed at investigating the onset timing of MAEs in cases using statin monotherapy and if concomitant drugs shifted the onset timing of MAEs.

## 2 MATERIALS AND METHODS

### 2.1 Data sources

The FAERS database Quarterly Data Files (Q1 2004 to Q3 2017) published by the FDA (downloaded in February 2018) was used to evaluate the adverse events associated with statin therapy. The Quarterly Data Files comprise 7 types of datasets (patient demographic and administrative information, DEMO; drug/biologic information, DRUG; adverse events, REAC; patient outcomes, OUTC; report sources, RPSR; drug therapy start and end dates, THER; and indication for use/diagnosis, INDI). Of these, DEMO, DRUG, REAC, and THER files were used for analyses.

### 2.2 Definition of MAEs

The following events were considered as MAEs based on a previous report^21^ taken from the Medical Dictionary for Regulatory Activities (MedDRA, version 20.1) at the Preferred Term level: “rhabdomyolysis” (MedDRA code 10039020), “myalgia” (10028411), “myoglobinuria” (10028629), and “blood creatine phosphokinase increased” (10005470).

### 2.3 Standardisation of names of drugs reported to FAERS

The drugs reported to FAERS can be registered by arbitrary names, including trade names and typographical errors.^22^ Therefore, we used DRUGBANK (version 5.0.11) to standardise the names of drugs, including statins and other concomitant drugs.^23^

### 2.4 Data extraction

**Figure 1:**
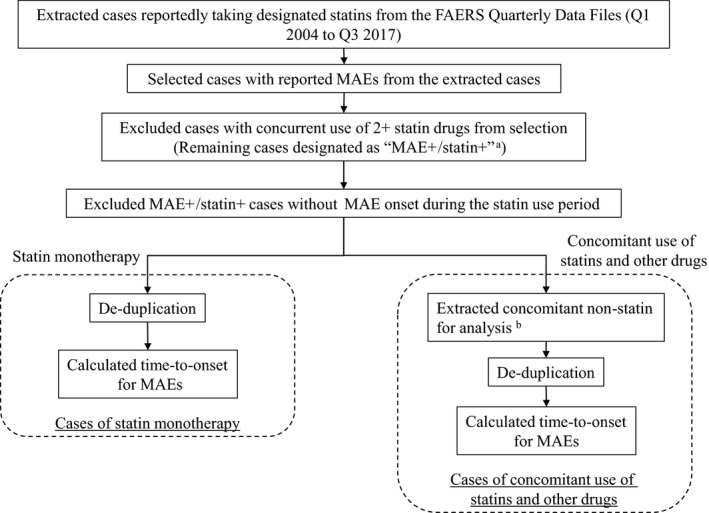
Flowchart for MAE time‐to‐onset calculation. (a) I.e., cases who received a statin drug and experienced MAEs. (b) I.e., non‐statin drugs whose use period overlapped with that of the designated statins. MAE, musculoskeletal adverse event

Figure 1 contains a flowchart depicting the study procedure from the extraction of cases reported in the FAERS to the calculation of MAE onset timing.

From the FAERS Quarterly Data Files, only cases in which atorvastatin, rosuvastatin, simvastatin, lovastatin, fluvastatin, pitavastatin, and pravastatin were prescribed were extracted for analysis. These selected cases were then condensed to cases in which the start date of statin use had been accurately recorded. Condensed cases with the recorded use of two or more statins were excluded, and the remaining cases were categorised as statin+ cases. Only cases who received a statin and experienced MAEs (ie MAE+/statin+ cases) were extracted. Among MAE+/statin+ cases, those cases in which MAEs developed after statin discontinuation or cases in which MAEs developed before the start of statin use were excluded. Finally, the MAE+/statin+ cases were divided into cases of statin monotherapy and cases of concomitant use of a specific statin and other drugs.

### 2.5 Cases of statin monotherapy

Some of the cases reported to the FAERS were the same cases that were reported by different reporters (duplicate cases). Thus, to exclude duplicate cases from our analysis, among the cases of statin monotherapy reported, cases in which all the 4 items of age, sex, adverse event onset date (EVENT_DT), and start date of statin use (START_DT), were the same were regarded as duplicate cases and eliminated. The differences between EVENT_DT and START_DT of cases of statin monotherapy were regarding the time‐to‐onset for MAEs. As the onset period (time‐to‐onset) of MAEs during statin use was mostly within 1 year,^10^ cases of statin monotherapy in which MAEs had developed within 365 days were selected for analysis in this study. A statistical analysis was performed to determine whether the time‐to‐onset of MAEs differed with statin type. In addition, statins for which less than 30 cases were reported for analysis were not included in the subsequent study in which the impact of concomitant drugs on the time‐to‐onset of MAEs was investigated (described below).

### 2.6 Cases of concomitant use of a specific statin and other drugs

**Figure 2:**
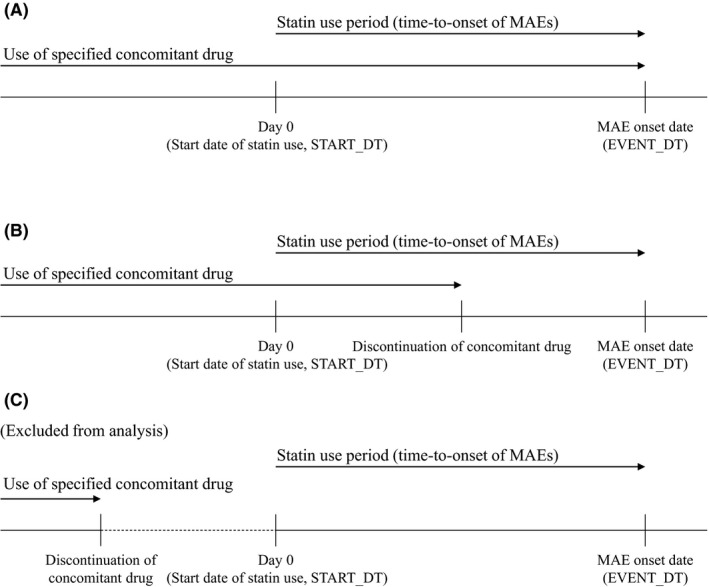
Selection of concomitant drugs for analysis. MAE, musculoskeletal adverse event

“Concomitant use of a specific statin and other drugs” here is defined as the concurrent administration of a specific statin and at least one type of nonstatin drug. The nonstatin drug(s) would need to be taken during the use period of the specific statin to investigate whether they can shift the onset timing of statin‐induced MAEs. If the statin use period and the use period of specified concomitant drugs (nonstatin) overlap, the risk of drug interactions may increase. Therefore, in cases of concomitant use of a specific statin and other drugs, nonstatin concomitant drugs used concurrently during statin use were selected for analysis (Figure 2A and B), while concomitant drugs that had been discontinued before the start date of statin use (START_DT) were excluded from the analysis (Figure 2C).

Among the reported cases of concomitant use, cases in which all the 4 items of age, sex, adverse event onset date (EVENT_DT), and the start date of statin use (START_DT) were the same were regarded as duplicate cases and thus deleted.

The differences between EVENT_DT and START_DT of the cases of concomitant use of a specific statin and other drugs were regarding the time‐to‐onset for MAEs, and these cases in which MAEs had developed within 365 days were selected for analysis. This procedure is the same as “Cases of statin monotherapy”. A statistical analysis was performed for concomitant drugs with more than 30 cases to determine whether the concomitant drugs changed the time‐to‐onset of MAEs.

### 2.7 Statistical analysis

Cases of statin monotherapy were collected and a nonparametric method, the Steel‐Dwass test, was used to determine if the time‐to‐onset of MAEs differed by statin type. This nonparametric analysis method was adopted as it was assumed that the time‐to‐onset would not be normally distributed. To determine if concomitant drugs affected the time‐to‐onset of statin‐induced MAEs, paired comparisons between cases of each concomitant drug use and cases of statin monotherapy were also performed using the Steel test.

All statistical analyses were performed using R software (version 3.2.2, R Foundation for Statistical Computing, Vienna, Austria) for Windows^®^. The significance level (*P*) was set at 0.05.

## 3 RESULTS

### 3.1 Time‐to‐onset of MAEs in cases of statin monotherapy

Table 1 shows the number of cases of statin monotherapy and the time‐to‐onset of MAEs. Among cases of atorvastatin monotherapy, 454 cases for which the time‐to‐onset could be calculated were selected for analysis. Among the seven types of statins, the number of cases of atorvastatin monotherapy was the largest followed by rosuvastatin (413 cases), simvastatin (409 cases), pravastatin (82 cases), lovastatin (34 cases), fluvastatin (29 cases), and pitavastatin (16 cases) in this study. The minimum time‐to‐onset for MAEs was 0.0 days (immediately after use) regardless of the statin type. The statin with the shortest median time‐to‐onset for MAEs was pitavastatin (14.0 days), followed by atorvastatin (24.5 days), rosuvastatin (30.0 days), simvastatin (38.0 days), pravastatin (43.0 days), fluvastatin (45.0 days), and lovastatin (48.0 days).

**Table 1:** Comparison of the onset timing of MAEs induced by each statin

Statin	No. of cases	MAE onset (days)					IQR
		Minimum value	First quartile	Median	Third quartile	Maximum value	
Atorvastatin^a^	454	0.0	0.0	24.5	78.8	361.0	0.0‐78.8
Rosuvastatin^b^	413	0.0	1.0	30.0	92.0	364.0	1.0‐92.0
Simvastatin^a^^,^ b	409	0.0	7.0	38.0	122.0	363.0	7.0‐122.0
Pravastatin	82	0.0	7.3	43.0	113.0	330.0	7.3‐113.0
Lovastatin	34	0.0	0.0	48.0	70.0	325.0	0.0‐70.0
Fluvastatin	29	0.0	21.0	45.0	112.0	300.0	21.0‐112.0
Pitavastatin	16	0.0	0.0	14.0	68.3	258.0	0.0‐68.3

Steel‐Dwass test. IQR, interquartile range; MAEs, musculoskeletal adverse events.

a Compared with simvastatin, atorvastatin was associated with a significantly faster onset of MAEs (*P* < 0.01).

b Compared with simvastatin, rosuvastatin was associated with a significantly faster onset of MAEs (*P* < 0.05).

The Steel‐Dwass test was performed to examine if the time‐to‐onset of MAEs differed by statin type. The test showed that the onset of MAEs induced by atorvastatin was significantly faster than that of MAEs induced by simvastatin (*P* < 0.01, median: 24.5 days vs 38.0 days). As in the case of atorvastatin, the onset of MAEs induced by rosuvastatin was significantly faster than that of simvastatin (*P* < 0.05, median: 30.0 days vs 38.0 days). However, the time‐to‐onset of MAEs induced by atorvastatin and rosuvastatin was not significant (*P* = 0.39, median: 24.5 days vs 30.0 days). The difference in the time‐to‐onset of MAEs could not be detected for statins with a small number of cases.

### 3.2 Concomitant drug‐associated shift in the onset timing of statin‐induced MAEs

Table 2 shows the time‐to‐onset of MAEs induced by atorvastatin and concomitant drugs. Twenty‐four different individual drugs were used concurrently with atorvastatin at a high frequency (≥30 cases), and the most frequently used concomitant drug was aspirin (299 cases). Compared to atorvastatin monotherapy, the concomitant drug that resulted in the shortest time‐to‐onset of MAEs was lisinopril (79 cases), with a median of 3.0 days and IQR of 0.0‐61.0 days. In contrast, the concomitant drug that resulted in the longest time‐to‐onset of MAEs was losartan (45 cases), with a median and IQR of time‐to‐onset for MAEs of 74.0 days and 7.0‐125.0 days, respectively. However, compared to atorvastatin monotherapy, these 24 concomitant drugs did not change the time‐to‐onset of MAEs significantly.

**Table 2:** Onset timing of MAEs induced by atorvastatin and concomitant drugs

Concomitant drugs	No. of cases (≥30 cases)	MAE onset (days)					IQR
		Minimum value	First quartile	Median	Third quartile	Maximum value	
Lisinopril	79	0.0	0.0	3.0	61.0	276.0	0.0‐61.0
Valsartan	45	0.0	0.0	5.0	41.0	261.0	0.0‐41.0
Atenolol	76	0.0	0.0	5.5	67.5	326.0	0.0‐67.5
Metoprolol	116	0.0	0.0	7.5	62.0	359.0	0.0‐62.0
Levothyroxine	92	0.0	0.0	8.5	92.0	334.0	0.0‐92.0
Acetaminophen	63	0.0	0.0	9.0	60.0	297.0	0.0‐60.0
Hydrochlorothiazide	78	0.0	0.0	9.0	71.5	297.0	0.0‐71.5
Omeprazole	84	0.0	0.0	9.0	107.3	350.0	0.0‐107.3
Diltiazem	35	0.0	1.0	11.0	36.5	335.0	1.0‐36.5
Fluticasone	37	0.0	0.0	11.0	151.0	294.0	0.0‐151.0
Warfarin	34	0.0	0.3	12.5	144.5	334.0	0.3‐144.5
Metformin	67	0.0	0.0	13.0	92.0	294.0	0.0‐92.0
Pantoprazole	37	0.0	1.0	13.0	42.0	335.0	1.0‐42.0
Aspirin	299	0.0	0.0	18.0	93.0	359.0	0.0‐93.0
Furosemide	89	0.0	1.0	18.0	90.0	322.0	1.0‐90.0
Ramipril	56	0.0	0.0	21.0	96.0	319.0	0.0‐96.0
Clopidogrel	112	0.0	1.0	22.0	86.0	350.0	1.0‐86.0
Salbutamol	36	0.0	1.0	22.0	100.8	294.0	1.0‐100.8
Ezetimibe	31	0.0	0.0	24.0	62.0	242.0	0.0‐62.0
Amlodipine	124	0.0	4.5	35.5	103.3	341.0	4.5‐103.3
Allopurinol	41	0.0	3.0	39.0	180.0	319.0	3.0‐180.0
Lansoprazole	42	0.0	1.0	39.0	142.0	304.0	1.0‐142.0
Bisoprolol	60	0.0	0.0	45.0	93.3	350.0	0.0‐93.3
Candesartan	34	0.0	5.3	52.0	190.5	347.0	5.3‐190.5
Losartan	45	0.0	7.0	74.0	125.0	334.0	7.0‐125.0

The Steel test was performed for cases of the atorvastatin monotherapy group (n = 454) as the control group. IQR, interquartile range; MAEs, musculoskeletal adverse events.

Table 3 shows the time‐to‐onset of MAEs induced by rosuvastatin and concomitant drugs. Twenty‐one concomitant drugs were used concurrently with rosuvastatin at a high frequency, and the most frequently used concomitant drug was aspirin (260 cases). The concomitant drug that resulted in the shortest time‐to‐onset of MAEs was ramipril (34 cases), with a median and IQR of time‐to‐onset for MAEs of 12.0 and 0.0‐103.0 days, respectively. In contrast, the concomitant drug that resulted in the longest time‐to‐onset of MAEs was furosemide (65 cases), with a median and IQR of time‐to‐onset for MAEs of 62.5 and 0.5‐144.0 days, respectively. Nevertheless, compared to rosuvastatin monotherapy, the 21 concomitant drugs did not change the time‐to‐onset of MAEs significantly.

**Table 3:** Onset timing of MAEs induced by rosuvastatin and concomitant drugs

Concomitant drugs	No. of cases (≥30 cases)	MAE onset (days)					IQR
		Minimum value	First quartile	Median	Third quartile	Maximum value	
Ramipril	34	0.0	0.0	12.0	103.0	212.0	0.0‐103.0
Olmesartan	33	0.0	3.0	19.0	71.0	275.0	3.0‐71.0
Lansoprazole	30	0.0	0.3	25.0	147.8	357.0	0.3‐147.8
Clopidogrel	94	0.0	1.0	30.0	120.0	363.0	1.0‐120.0
Warfarin	46	0.0	1.3	30.0	69.0	317.0	1.3‐69.0
Aspirin	260	0.0	1.0	31.0	92.0	334.0	1.0‐92.0
Atenolol	61	0.0	0.0	31.0	78.0	362.0	0.0‐78.0
Levothyroxine	92	0.0	7.0	31.0	102.3	363.0	7.0‐102.3
Candesartan	31	0.0	0.5	32.0	75.5	282.0	0.5‐75.5
Lisinopril	79	0.0	1.5	34.0	115.5	363.0	1.5‐115.5
Omeprazole	90	0.0	2.3	34.5	125.5	314.0	2.3‐125.5
Metformin	64	0.0	0.0	36.0	105.0	334.0	0.0‐105.0
Acetaminophen	43	0.0	1.0	37.0	101.0	286.0	1.0‐101.0
Metoprolol	97	0.0	1.0	38.0	148.0	349.0	1.0‐148.0
Bisoprolol	35	0.0	10.5	40.0	113.0	334.0	10.5‐113.0
Valsartan	54	0.0	2.3	40.5	143.5	359.0	2.3‐143.5
Hydrochlorothiazide	84	0.0	2.8	45.5	116.0	334.0	2.8‐116.0
Amlodipine	89	0.0	5.0	47.0	116.0	363.0	5.0‐116.0
Esomeprazole	49	0.0	1.0	47.0	224.0	314.0	1.0‐224.0
Ezetimibe	44	0.0	0.8	50.0	117.5	258.0	0.8‐117.5
Furosemide	65	0.0	10.0	55.0	121.0	302.0	10.0‐121.0
Fluticasone	30	0.0	0.5	62.5	144.0	353.0	0.5‐144.0

The Steel test was performed for cases of the rosuvastatin monotherapy group (n = 413) as the control group. IQR, interquartile range; MAEs, musculoskeletal adverse events.

Table 4 shows the time‐to‐onset of MAEs induced by simvastatin and concomitant drugs. Twenty‐six concomitant drugs were used concurrently with simvastatin at a high frequency, and the most frequently used concomitant drug was aspirin (249 cases). The concomitant drug that resulted in the shortest time‐to‐onset of MAEs was amlodipine (93 cases), with a median and IQR of time‐to‐onset for MAEs of 12.5 and 0.0‐73.8 days, respectively. In contrast, the concomitant drug that resulted in the longest time‐to‐onset of MAEs was nitroglycerin (42 cases), with a median and IQR of time‐to‐onset for MAEs of 64.5 and 23.0‐197.3 days, respectively. Nonetheless, compared to simvastatin monotherapy, all these 26 concomitant drugs did not change the time‐to‐onset of MAEs significantly.

**Table 4:** Onset timing of MAEs induced by simvastatin and concomitant drugs

Concomitant drugs	No. of cases (≥30 cases)	MAE onset (days)					IQR
		Minimum value	First quartile	Median	Third quartile	Maximum value	
Fluticasone	34	0.0	0.0	12.5	73.8	250.0	0.0‐73.8
Amlodipine	93	0.0	1.0	20.0	91.0	334.0	1.0‐91.0
Isosorbide Mononitrate	37	0.0	12.0	26.0	69.0	290.0	12.0‐69.0
Atenolol	74	0.0	0.8	26.5	90.5	313.0	0.8‐90.5
Diltiazem	56	0.0	5.5	26.5	73.5	335.0	5.5‐73.5
Losartan	42	0.0	0.5	27.5	109.3	351.0	0.5‐109.3
Clopidogrel	115	0.0	5.5	29.0	91.0	362.0	5.5‐91.0
Ramipril	68	0.0	10.3	29.0	77.0	301.0	10.3‐77.0
Aspirin	249	0.0	3.0	30.0	99.0	364.0	3.0‐99.0
Furosemide	107	0.0	4.0	30.0	76.0	351.0	4.0‐76.0
Metoprolol	102	0.0	5.3	30.0	117.3	364.0	5.3‐117.3
Omeprazole	110	0.0	4.0	30.5	73.0	333.0	4.0‐73.0
Salbutamol	46	0.0	2.0	30.5	98.0	325.0	2.0‐98.0
Levothyroxine	75	0.0	5.0	31.0	127.5	352.0	5.0‐127.5
Pantoprazole	35	0.0	5.5	31.0	185.0	301.0	5.5‐185.0
Hydrochlorothiazide	68	0.0	0.0	33.0	153.5	338.0	0.0‐153.5
Metformin	84	0.0	9.0	33.5	130.8	364.0	9.0‐130.8
Allopurinol	47	0.0	16.5	34.0	108.0	263.0	16.5‐108.0
Lisinopril	91	0.0	4.0	34.0	121.0	335.0	4.0‐121.0
Cyclosporine	38	0.0	14.5	34.5	94.8	352.0	14.5‐94.8
Lansoprazole	37	0.0	12.0	37.0	202.0	364.0	12.0‐202.0
Warfarin	43	0.0	12.0	41.0	165.5	303.0	12.0‐165.5
Bisoprolol	56	0.0	13.0	43.5	172.5	352.0	13.0‐172.5
Gemfibrozil	59	0.0	30.5	45.0	114.5	351.0	30.5‐114.5
Ezetimibe	30	0.0	19.3	52.0	95.8	335.0	19.3‐95.8
Acetaminophen	71	0.0	6.0	61.0	142.0	333.0	6.0‐142.0
Nitroglycerin	42	0.0	23.0	64.5	197.3	351.0	23.0‐197.3

The Steel test was performed for cases of the simvastatin monotherapy group (n = 409) as the control group. IQR, interquartile range; MAEs, musculoskeletal adverse events.

Aspirin was the only concomitant drug used concurrently with pravastatin at a high frequency (30 cases, median: 31.5 days; IQR: 4.0‐193.0 days), and compared to pravastatin monotherapy, its use did not change the time‐to‐onset of MAEs significantly.

## 4 DISCUSSION

### 4.1 Onset timing of MAEs by statin type

The maximum number of cases of MAE onset was associated with atorvastatin, followed by rosuvastatin and simvastatin, and the number of cases exceeded 400 for the three statins. The number of MAE cases induced by pravastatin, lovastatin, fluvastatin, and pitavastatin was small. In particular, only 16 cases were extracted for pitavastatin. The onset timing for MAEs (median) induced by atorvastatin and rosuvastatin was respectively 24.5 and 30.0 days, which was significantly shorter than those associated with simvastatin (43.0 days). The onset timing for MAEs induced by pravastatin, lovastatin, and fluvastatin were similar to simvastatin at generally 40 days.

The magnitude of HMG‐CoA reductase 50% inhibitory concentration (IC_50_) for each statin was in the order of rosuvastatin < atorvastatin < simvastatin < fluvastatin < pravastatin.^24^ Atorvastatin or rosuvastatin, which possesses a high HMG‐CoA reductase inhibitory activity, is considered high‐intensity statin therapy that reduces LDL cholesterol by more than 50%. The use of the other statins is considered either moderate or low‐intensity statin therapy.^1^ Hoffman et al reported the following order for the relative risk of statin‐induced MAEs: rosuvastatin > atorvastatin > simvastatin > pravastatin > lovastatin.^25^ These indicate that the higher the HMG‐CoA reductase inhibitory activity (or the lower the IC_50_) of a statin, the higher the relative risk of MAEs tends to be. Previous study findings and the results of the present study suggest that statins with a high HMG‐CoA reductase inhibitory activity, such as atorvastatin and rosuvastatin, not only increase the onset risk of MAEs, but also induce MAEs within a short time.

We also considered the relationship between statin lipophilicity and MAE onset timing. Rosuvastatin and pravastatin are known to have very low lipophilicity than other statins (in descending order: simvastatin > fluvastatin > atorvastatin > rosuvastatin > pravastatin).^24^ Statin lipophilicity does not correlate with the relative risk of statin‐induced MAEs nor the MAE onset timing for any statin. Thus, it is unlikely that MAE risk and timing are affected by statin lipophilicity.

Thus, our research finding that the onset timing of MAEs differs with statin type is important for reducing the risk of side effects and side effect prevention in a clinical setting. The onset timing for MAEs in cases of pitavastatin use was extremely short. This might be due to the very small number of cases (only 16) of pitavastatin use. This could be attributable to the relatively recent approval of pitavastatin (in 2009) compared to other statins and to its low prescription rate.^26^, ^27^ Thus, a more accurate onset timing for MAEs may be determined in the future if spontaneous case reports continue to accumulate in the FAERS.

### 4.2 Effects of concomitant drugs on the onset timing of MAEs

This study also investigated whether concomitant drugs used concurrently with statins impacted the onset timing of MAEs. The results showed that concomitant drugs in all statin‐concomitant drug combinations evaluated in this study did not affect the onset timing of MAEs.

Atorvastatin is mainly metabolised by CYP3A4 and partially by CYP2C8.^28^, ^29^ Similar to atorvastatin, 1,4‐dihydropyridine calcium channel blockers such as amlodipine are metabolised by CYP3A4; therefore, the MAE onset risk may increase because of DDI.^30^ However, the blood concentration is not greatly affected, and the effect is not clinically significant.^31^ Similar to amlodipine, diltiazem is metabolised by CYP3A4, and the MAE onset risk may increase.^30^, ^32^ Nevertheless, the concomitant use of atorvastatin and diltiazem did not cause changes in the onset timing of MAEs (Table 2).

Simvastatin is mostly metabolised by CYP3A4, while some of it is metabolised by CYP2C8.^33^, ^34^ It has been known that the concomitant use of simvastatin and amlodipine increases the risk of myopathy.^35^, ^36^ In addition, the concomitant use of simvastatin and cyclosporine is contraindicated as cyclosporine inhibits CYP3A4.^37^ However, these drugs did not cause changes in the onset timing of MAEs.

Thus, it was clarified that even if the concomitant use of statins and drugs that may cause DDI can change the MAE onset risk, it is unlikely that the onset timing will be changed. The results also showed that it is unlikely for other concomitant drugs, which are thought to have no drug interaction with statins, to cause changes in the onset timing of MAEs.

### 4.3 Limitations

This study has certain limitations. (1) This study did not include cases reported before 2004, because only data from the year 2004 was available for download from FAERS. Therefore, cases of statin use before 2001, which had been analysed by Chang et al, could not be evaluated in this study, and the number of cases reported for some statins was insufficient. (2) This study considered only four types of adverse events as MAEs for analysis: rhabdomyolysis, myoglobinuria, myalgia, and blood creatine phosphokinase increased. The latter two are common side effects of statins; however, this does not mean that statins are necessarily the cause of these adverse events when they do occur. Moreover, the magnitude of creatine phosphokinase increase (ie, in terms of laboratory test values) was not reported in the records of cases receiving statins in the FAERS database. Accordingly, our data cannot be interpreted to conclusively prove that any of the four phenotypes defined here were caused by statins in the analysed cases with concomitant nonstatin drugs. (3) Statin dosage is related to the intensity of LDL cholesterol‐lowering effect. For example, a daily dose of rosuvastatin 20 mg is considered a high‐intensity statin therapy, while a daily dose of rosuvastatin 10 mg is a moderate‐intensity statin therapy.^1^ Therefore, if the intensity of statin therapy is related to the time‐to‐onset for MAEs, the dosage must be taken into consideration. However, in cases reported to FAERS, data regarding administration and dosage are often missing; thus, the statin dosage of each case could not be taken into consideration. Hence, the changes in onset timing of MAEs dependent on statin dosage could not be evaluated in this study.

## DISCLOSURES

The authors declare no conflict of interest.
